# Potyvirus-Based Vectors for Heterologous Gene Expression in Plants

**DOI:** 10.3390/v16121920

**Published:** 2024-12-15

**Authors:** Adam Achs, Miroslav Glasa, Zdeno Šubr

**Affiliations:** 1 Department of Virus Ecology, Institute of Virology, Biomedical Research Center of the Slovak Academy of Sciences, Dubravska Cesta 9, 845 05 Bratislava, Slovakia; 2 Faculty of Natural Sciences, University of Ss. Cyril and Methodius in Trnava, Námestie J. Herdu 2, 917 01 Trnava, Slovakia

**Keywords:** potyviruses, viral vectors, transient expression, recombinant proteins, plant viruses

## Abstract

Over the past two decades, plant viral vectors have emerged as a powerful tool for the production of recombinant proteins in plants. Among the different plant viruses engineered to carry foreign genes of interest in their genomes, potyviruses have gained attention due to their polyprotein expression strategy and broad host range. To date, at least eleven different species belonging to the genus *Potyvirus* have been used for heterologous gene expression in both their natural and experimental hosts. This review article provides an overview of the current state of potyvirus-based plant viral vectors, discussing the advantages and limitations of these systems. We also discuss the future challenges and potential applications of potyvirus-based expression vectors, including the production of vaccines, nanoparticles, therapeutics, and metabolic engineering. Overall, we highlight the potential of potyvirus-based vectors as a versatile tool for recombinant protein production in plants.

## 1. Introduction

Plants offer a promising approach to the rapid, cost-effective, and scalable production of recombinant proteins. Plants are capable of performing a high level of post-translational modifications, such as glycosylation and phosphorylation, which are important to maintaining the biological activity and stability of many recombinant proteins [1]. Thus, they are able to produce complex proteins that are difficult to produce in other systems, such as bacteria or yeasts. Plant viral vectors have emerged as a powerful alternative to the use of transgenic plants for heterologous expression. Unlike transgenic approaches, which often involve time-consuming procedures for stable integration of foreign genes into the plant genome, plant viral vectors can rapidly introduce and express foreign genes in a wide range of plant species. These vectors exploit the natural infection mechanisms of viruses to deliver genes efficiently, resulting in high levels of protein expression within days of inoculation. Additionally, plant viral vectors circumvent issues related to stable integration into the plant genome, thereby reducing concerns over potential off-target effects and genetic instability [2]. This rapid, high-yield, and flexible approach has made plant viral vectors a valuable tool for research and biotechnological applications, such as the production of vaccines, therapeutic proteins, and other valuable biomolecules [3].

**Figure 1:**
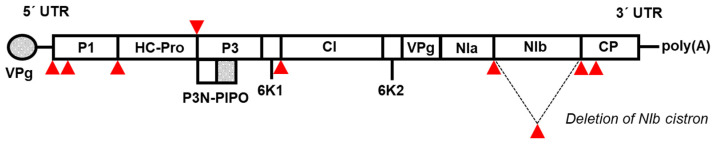
Overview of insertion sites within the potyviral genome used for the incorporation of foreign genes. The red arrows highlight nine insertion sites where foreign genes have been successfully incorporated and tested. While the P1/HC-Pro and NIb/CP junctions have been the primary insertion sites in most studies, additional positions have been tested, including the HC-Pro/P3 and 6K1/CI junctions [8], the 5′ UTR and P1 cistron [9], and internal sites within the N-termini of the P1 [10] and CP [11] cistrons. Moreover, the insertion site between the *NIa* and *CP* cistrons has been employed by replacing the *NIb* cistron with a foreign sequence, with the NIb protein supplied in trans for functional complementation [12].

The genus *Potyvirus* (family *Potyviridae*) is the largest among plant viruses and includes many economically significant pathogens of crop plants. These are filamentous viruses with a (+)ssRNA genome approximately 10 kb in length, which includes a single ORF encoding a precursor polyprotein (Figure 1). This polyprotein is co- and post-translationally cleaved by three viral proteases into 11 gene products, including the P3N-PIPO protein, which arises from an alternative ORF within the *P3* cistron due to a translational frameshift [4,5]. The biotechnological potential of potyviruses is determined by several of their properties. The polyprotein strategy of gene expression ensures the production of the target protein in an equimolar ratio to other viral proteins. Thus, its accumulation rate depends solely on its stability and the intrinsic replication of the virus. By flanking the target protein with specific cleavage sites for the viral protease, it is possible to achieve efficient cleavage from the polyprotein precursor and hence its accumulation in a free form. Introducing additional insertion sites within the genome allows for the co-expression of multiple genes from a single vector simultaneously [6]. To date, nine insertion sites have been successfully tested and are further discussed in this article (Figure 1). Due to the filamentous morphology of potyvirus particles, the resulting size of the incorporated genome is not as strictly limited as in viruses with icosahedral symmetry. To date, at least 206 different potyviruses have been identified (according to an ICTV report, 24 October 2024). Given this extraordinary diversity of potyvirus species, potyviruses have a broad host range in which heterologous expression can be induced [7]. Several potyvirus species have been used for this purpose so far. Here, we provide a comprehensive overview of particular potyviruses used for heterologous expression in plants.

## 2. Potyvirus-Mediated Expression of Foreign Genes in Plants

### 2.1. Tobacco Etch Virus

Tobacco etch virus (TEV, *Potyvirus nicotianainsculpentis*, based on the ICTV binomial classification) was the first potyvirus used for the expression of a foreign gene. The full-length cDNA clone of TEV was modified by inserting a gene encoding GUS (*uidA*) at the 5′ end of the HC-Pro gene. The result was the expression of enzymatically active fusion forms of GUS fused with HC-Pro, allowing for histochemical in situ visualization of virus replication in various tobacco tissue types. The modified virus retained its ability to spread systemically, but its accumulation was lower compared to the wild-type TEV, and the infected plants exhibited milder symptoms. The construct was genetically stable over several rapid mechanical passages, but spontaneous deletions in the insert area and adjacent regions of the TEV genome were observed during prolonged cultivation of infected plants [13,14]. Subsequent research achieved the expression of free GUS by introducing an additional cleavage site for NIa protease between the sequences encoding GUS and HC-Pro [15]. These experiments provided the first evidence that potyviruses could serve as vectors for the expression of free polypeptides in planta. Attention was also given to elucidating the probable mechanism of spontaneous deletion mutations. Similarly to related RNA virus taxa, potyviruses are presumed to involve non-homologous recombination, which occurs due to template switching after the dissociation of the transcriptase complex from the original template strand during synthesis (the so-called copy choice mechanism). Non-homologous recombination can occur between two genetically distinct regions, leading to the emergence of deletion genotypes with subsequent selection of viable or advantageous variants [14,16].

Based on previous experiments, the pTEV-NH vector was prepared. It includes an additional site for NIa-Pro, as well as a polylinker and a hexahistidine tag for N-terminal fusion with the target protein [17]. The resulting vector was used to characterize selected proteins of Beet yellows virus (BYV), p20, HSP70h, L-Pro, and CP via infection of *Nicotiana tabacum* or *Nicotiana benthamiana*. The individual closterovirus proteins showed varying impacts on the course of infection, virus accumulation, and symptom manifestation. In the case of spontaneous insert deletions, adjacent parts of HC-Pro were also removed, but not P1, indicating the essential role of this gene during viral infection. Analysis of the model of *N. tabacum* protoplasts transfected with the constructs did not show an impact of the inserted genes on TEV genome replication or encapsidation at the cell level. Thus, the influence on plant infection likely occurred at the level of systemic virus–host interaction, e.g., by affecting plant defense mechanisms or systemic virus spread [18].

In the following years, the binary plasmid pGTEV was prepared, consisting of the complete TEV genome flanked by the 35S promoter from Cauliflower mosaic virus (CaMV) and CaMV terminator, cloned into a binary vector derived from plasmid pCLEAN-G181. This allowed for efficient infection of host plants via agroinfiltration [19]. pGTEV was used for the construction of the defective vector pGTEVΔNIb, where the cistron encoding NIb was replaced with the corresponding expression cassette (Figure 1), with NIb being complemented in trans via transgenesis or co-infection with a compatible NIb-expressing potato virus X-based vector (PVX-NIb). Partial deconstruction of the TEV genome enabled not only individual expression but also simultaneous co-expression of three labeled reporter fluorescent proteins, FLAG-mCherry, HA-Venus, and c-Myc-mTagBFP, flanked by cleavage sites for NIa-Pro protease. Transgenic *N. tabacum* with constitutive NIb expression and *N. benthamiana* co-infected with PVX-NIb were infected with the constructs. While individual expression of the proteins was successful through both methods of NIb complementation, co-expression in the presence of PVX-NIb could not be achieved. Constructs for individual expression showed genetic stability even after mechanical virus passage, while RT-PCR analysis of the co-expression construct showed the presence of deletion variants. Subsequent experiments with the pGTEVΔNIb vector focused on its use in manipulating plant metabolism. A construct for co-expression of transcription factors Delila and Rosea1 from *Antirrhinum majus*, responsible for activating anthocyanin biosynthesis, was prepared. Infection of transgenic *N. tabacum* with this construct led to the production of these pigments in symptomatic leaves, resulting in tissue color changes [12].

Given the successful equimolar co-expression of multiple proteins from a single pGTEVΔNIb construct, further experiments focused on the possibility of their individual translocation to different cellular compartments. A series of constructs for GFP expression with an insertion site between the 5′ UTR and P1 cistron (N-terminal position) or between the *NIa-Pro* and *CP* genes, i.e., in place of the NIb gene (internal position), were prepared. The inserted GFP-encoding gene was also fused at the N-terminus with translocation signals for transportation to chloroplasts, the nucleus, and mitochondria. While GFP translocation to chloroplasts and mitochondria occurred only in the case of its N-terminal insertion, nuclear localization of GFP was successful in both polyprotein positions. This can be attributed to the tertiary structure of the viral polyprotein before cleavage, which, in the case of internal GFP localization, may prevent the interaction of signal sequences with the cell translocation machinery proteins. Amino acid residues from the NIa-Pro cleavage site may also interfere with signal peptide recognition [9]. Unlike chloroplasts and mitochondria, nuclear translocation allows for transportation of fully folded proteins, occurring through large nuclear pores. It is assumed that the nuclear translocation signal creates surface loops that enable protein recognition even in the folded state [20]. Based on these findings, a construct for N-terminal GFP expression with chloroplast translocation and internal mCherry expression with nuclear translocation was prepared. Both proteins were successfully detected in target structures, demonstrating that the pGTEVΔNIb vector can be used for the co-expression of two different proteins and their transportation to different cell organelles. Western blot analysis of infected tissues also showed a lower accumulation rate of GFP in the case of mitochondrial translocation, suggesting that some signal peptides may negatively affect the final yield. Differences were also noted between the various GFP positions within the viral polyprotein. Constructs with internal GFP localization showed higher genetic stability, while RT-PCR products of a smaller size, indicating GFP cistron deletion, were detected in the case of N-terminal GFP insertion [9].

The vector pGTEVΔNIb was further used to express a whole multistep heterologous metabolic pathway in tobacco plants to synthetize lycopene, a health-promoting carotenoid. The pathway involved three genes from the soil bacterium *Pantoea ananatis*, *crtE*, *crtB*, and *crtI,* encoding enzymes required for lycopene biosynthesis. All three genes were inserted into the pGTEVΔNIb. Tobacco plants infected with the resultant construct exhibited distinct red spots on their leaves, indicating lycopene accumulation, which was later confirmed through HPLC analysis. Analysis of RNA confirmed successful expression of all three genes in these tissues. Lycopene levels reached approximately 10% of total carotenoid content, equating to around 150 μg per gram of dry weight, demonstrating the potential of this system for the synthesis of bioactive compounds in plants. To further confirm that lycopene production required the complete biosynthetic pathway, three individual clones expressing each biosynthetic enzyme were constructed. Interestingly, *crtB* expressing plants exhibited a striking yellow coloration by 10 days post inoculation (dpi). HPLC analysis revealed that the yellow tissues had elevated levels of chloroplastic carotenoids along with a reduction in chlorophyll content. The combination of viral-infection-induced chlorosis and increased carotenoid accumulation likely contributed to the yellow pigmentation. These findings suggested that the *crtB* gene could serve as a visual reporter for tracking viral infection in plants. Indeed, it was successfully used to track viral infection in *N. benthamiana*, *Solanum lycopersicum*, *Arabidopsis thaliana,* and *C. pepo* [21].

### 2.2. Plum Pox Virus

Plum pox virus (PPV, *Potyvirus plumpoxi*) is one of the most significant pathogens of fruit trees in the genus *Prunus*, causing the disease known as sharka. Its natural host range includes several economically important species, such as European plum (*P. domestica*), peach (*P. persica*), sour cherry (*P. cerasus*), cherry plum (*P. cerasifera*), and almond (*P. dulcis*). The experimental host range of PPV includes various herbaceous species across different families, including *N. benthamiana* (as well as other *Nicotiana* species), *Chenopodium foetidum*, *P. sativum*, and opium poppy (*Papaver somniferum*) [22,23,24].

For heterologous expression purposes, an infectious clone pGPPV was initially modified. This clone consisted of the full-length cDNA of the PPV-D strain (Rankovic isolate) under the control of the T7 bacteriophage promoter [25]. A cloning site was introduced between the *P1* and *HC-Pro* genes. The GUS gene, including a recognition site for NIa-Pro, was cloned into the resulting vector. Infection developed in 40–50% of inoculated *N. clevelandii* plants (100% for pGPPV) during two independent experiments, and the infected plants did not exhibit any symptoms. Subsequent analysis showed significantly slower but comparable accumulation of the modified virus in later stages of infection compared to the parental pGPPV. The infection kinetics matched the GUS activity in infected leaves. The construct was stable in primarily infected plants even at 52 dpi, but extensive deletions in the insert area were observed after the first mechanical passage of the virus [26]. In subsequent experiments, an infectious clone pICPPV was prepared from pGPPV. The T7 bacteriophage promoter was replaced by the CaMV 35S promoter, and the nopaline synthase terminator was incorporated. To enhance cloning efficiency, an intron was introduced into the P3 cistron, which exhibited toxicity to *E. coli*, leading to faster bacterial growth and higher plasmid DNA yields [27]. The vector PPV-NK, derived from pICPPV, included an insertion cassette between the *NIb* and *CP* genes, consisting of a polylinker and an additional recognition site for NIa protease. Initially, the reporter gene for GFP was cloned into PPV-NK. Infected *N. clevelandii* exhibited symptoms comparable to the wild-type PPV, and immunochemical analysis demonstrated effective cleavage of GFP from the viral polyprotein. Expression of functional GFP in infected leaves was confirmed through fluorescence microscopy even before the onset of infection symptoms. Moreover, the construct was genetically stable for at least 30 dpi, even after mechanical passage. To test the vector’s utility for vaccine antigen production in plants, a construct for the expression of the VP60 structural protein of the Rabbit hemorrhagic disease virus (RHDV) was prepared. In this case, there was no reduction in infectivity or change in infection symptomatology compared to wild-type PPV, and VP60 expression was immunochemically detected in plants at 15 dpi. The presence of truncated forms of VP60 at 21 dpi indicated genetic instability of the construct, with RT-PCR analysis confirming partial deletions of the insert. However, full-length VP60 predominated in most infected plants. The expressed VP60 was administered subcutaneously to rabbits in two doses as a clarified extract from infected leaves combined with an oil adjuvant. Immunization induced a specific antibody response against RHDV, with complete protective efficacy against lethal infection [28].

Another example is the use of the full-length clone p35PPV-NAT [29], into which cistrons encoding the fluorescent proteins GFP and DsRed were inserted between the *NIb* and *CP* genes. The prepared constructs, along with other labeled potyviruses, allowed for monitoring their spatial distribution during single or mixed infection with PVX [30].

In our laboratory, an infectious clone of the PPV-Rec strain was prepared by gradually replacing the cDNA of PPV in pICPPV [27] with homologous regions from the BOR-3 isolate while retaining the intron inserted in the P3 cistron [31]. From the resulting clone pICPPV-Rec, the expression vector pAD (referred to as pBOR-VT in the older literature) was derived by reintroducing the cloning linker from pICPPV. To ensure the biological safety of the vector, mutagenesis of the DAG motif in the N-terminal region of CP, essential for aphid transmission of PPV, was performed [32,33]. The pAD vector was infectious after the insertion of various foreign genes, and it was genetically stable. Pilot experiments focused on expressing the PB1-F2 protein of influenza A virus (IAV) in *N. benthamiana*, but its detection and potential purification from infected plants were limited by its low solubility in planta [34]. In subsequent experiments, the cDNA of PPV from the pAD vector was recloned into the binary plasmid pCambia 1304 with a deleted GUS gene, enabling methodologically simpler and more efficient plant transfection through agroinfiltration. The resulting vector, designated as pAD-agro, was subsequently used for the expression of different foreign proteins in *N. benthamiana* [35]. Moreover, the susceptibility of several alternative hosts to the experimental agroinfection with pAD-agro was evaluated. Systemic PPV infection was successfully established in domestic plum (*P. domestica*) seedlings and opium poppy (*P. somniferum*) [24].

Initially, a sequence encoding a small heat-shock protein from the bacterium *Cronobacter sakazakii* (designated sHSPhis) was cloned into the pAD-agro vector, with a hexahistidine tag fused at both ends. The resulting construct, pAD-agro-sHSPhis, was infectious and remained stable even after prolonged cultivation. Accumulation of sHSPhis in plants was observed between 7 and 14 dpi, peaking at 9 dpi. However, low levels of sHSPhis were still detectable at 75 dpi. Interestingly, the detection signal was significantly higher in roots compared to leaves, suggesting greater stability of sHSPhis in root tissues. Both leaves and roots harvested at 9 dpi were used as source material for sHSPhis purification via immobilized metal affinity chromatography (IMAC). While extraction under native conditions was unsuccessful, the use of 6M guanidinium chloride effectively solubilized sHSPhis from leaves, yielding 24 μg per gram of fresh leaf tissue. Despite higher sHSPhis accumulation in roots, extraction efficiency from this tissue was lower, yielding about 8 μg per gram of fresh root tissue [35].

Subsequent experiments focused on the expression of the full-length nucleocapsid (N) protein of the SARS-CoV-2 virus, as well as its N- and C-proximal fragments, each approximately half of the size of the full protein. All three constructs systemically infected *N. benthamiana* plants and demonstrated long-term genetic stability. The N-proximal fragment exhibited a high accumulation rate in systemically infected leaves, reaching levels comparable to the PPV coat protein. The product was successfully purified by IMAC under denaturing conditions, yielding approximately 78 μg/g of fresh leaf tissue. In contrast, the full-length N protein and its C-proximal fragment showed signs of significant proteolytic degradation, resulting in low accumulation levels of these proteins [35].

### 2.3. Clover Yellow Vein Virus

Clover yellow vein virus (CYVV, *Potyvirus trifolii*) is a virus infecting many members of the *Fabaceae* family (legumes), including economically significant legumes, such as broad bean (*Vicia faba*), common bean (*Phaseolus vulgaris*), pea (*Pisum sativum*), and soybean (*Glycine max*) [36]. The backbone for the construction of an expression vector based on CYVV was its infectious cDNA clone pClYVV [37], into which the GFP gene, including an additional recognition site for NIa protease, was inserted between the *P1* and *HC-Pro* genes. The resulting construct, pClYVV-GFP, was subsequently used to infect broad bean, common bean, pea, and soybean, which developed typical mosaic symptoms (with necrotic symptoms in broad bean and common bean) comparable to the wild-type virus. In the case of *N. benthamiana* infection, the appearance of typical chlorotic spots was observed. GFP accumulation in broad beans reached 46 mg/kg of fresh leaf weight, nearly matching the concentration of CYVV CP in infected plants [36].

For practical application of the vector, the gene encoding soybean cytosolic glutamine synthetase (GS), involved in nodulation in legumes, was cloned into the pClYVV-GFP construct. The GS coding sequence was flanked by NIa protease recognition sites on both termini to ensure cleavage from the viral polyprotein. The resulting dual construct, pClYVV-GFP-GS, containing both GFP and GS coding genes, was systemically infectious in broad bean following inoculation, but without necrotic symptoms. Symptomatic leaves exhibited green fluorescence corresponding to GFP expression. Immunochemical analysis of infected tissues confirmed the presence of both proteins in a proteolytically processed form. RT-PCR analysis demonstrated the genetic stability of the construct for at least 2 months post-inoculation. The phenotypic effects of GS overexpression in planta were evaluated by incubating symptomatic leaves in the presence of glufosinate, a specific GS inhibitor. The tested leaves showed a certain level of resistance to glufosinate, as a delayed onset of its herbicidal effect was observed [36].

### 2.4. Lettuce Mosaic Virus

Lettuce mosaic virus (LMV, *Potyvirus lactucae*) is one of the most devastating viral pathogens affecting lettuce and other leafy vegetables. To monitor the spread of LMV in susceptible and resistant lettuce cultivars, reporter genes for GFP and GUS were inserted into the cDNA clone of its isolate LMV-E in an N-terminal fusion with HC-Pro. The resulting constructs, LMV-E-GFP and LMV-E-GUS, were used to infect lettuce plants (*Lactuca sativa*), which developed typical mosaic symptoms (with LMV-E-GUS showing a slight delay). However, the symptoms were milder compared to the parental virus and did not include stunting, leaf deformation, or necrotic lesions. Virus accumulation in systemically infected tissues was also reduced. The LMV-E-GFP construct was phenotypically stable for at least six passages based on GFP expression, but long-term (four-month) propagation within an individual host led to the loss of emitted fluorescence. For LMV-E-GUS, GUS activity was lost both after serial passage and during prolonged infection. Subsequent RT-PCR analysis demonstrated deletions in the inserted gene and the adjacent HC-Pro cistron [38].

### 2.5. Zucchini Yellow Mosaic Virus

Zucchini yellow mosaic virus (ZYMV, *Potyvirus cucurbitaflavitesselati*) is a globally widespread pathogen causing disease in members of the *Cucurbitaceae* family, which includes economically important crops, such as cucumber, squash, zucchini, and watermelon [39]. To develop a non-pathogenic and biologically safe expression vector, an attenuated ZYMV designated as AG, which does not cause phenotypic damage in infected plants, was used [40]. Targeted mutagenesis was performed in the AG genome by substituting alanine with threonine in the DAG motif of the CP, which is essential for virus transmission by aphids. An insertion cassette for cloning foreign genes in translational in-frame fusion with adjacent cleavage sites for P1 and NIaPro proteases was inserted between the *P1* and *HC-Pro* genes of the resulting vector. The genes for the cucumber mosaic virus (CMV) CP and the watermelon mosaic virus CP were inserted into this modified vector (designated as AGIII). Despite the infectivity of the prepared constructs, the expression of target proteins in plants was not observed. Subsequent RT-PCR analysis demonstrated partial deletion of the inserted segments [41].

Due to the apparent genetic instability of the AGIII vector, a vector AGII with an insertion site between the *NIb* and *CP* genes, flanked by two cleavage sites for NIa-Pro, was constructed. After inserting the gene for CMV CP into AGII, symptoms developed in several tested plant species, and Western blot analysis confirmed the expression of CMV CP in symptomatic tissues. The construct was also genetically stable. These results suggested that the position between the *NIb* and *CP* genes represents a more suitable area for the insertion of foreign genes. For a closer characterization of virus distribution in plants, reporter genes for GUS and GFP were inserted into AGII. Plants infected with the AGII-GFP construct exhibited typical symptoms starting 5–7 dpi. The construct was genetically stable even 24 dpi and after four mechanical passages. In contrast, plants infected with the AGII-GUS construct developed symptoms with a 4-day delay, and RT-PCR analysis confirmed deletions in the insert area as early as 14 dpi. The differences in stability of the constructs indicated their probable dependence on the size of the inserted genes (less than 800 bp for CMV CP and GFP and around 1800 bp for GUS) [41].

The biotechnological potential of the AGII vector was tested by inserting a gene encoding human interferon (IFN) α2. The prepared AGII-IFN construct was genetically stable even 2 months after plant inoculation and during at least six mechanical passages. Infection of cucumber and squash plants with the AGII-IFN construct led to the production of biologically active IFN-α2 in fruits and leaves. The highest activity of IFN-α2 was observed in leaves, while its activity in fruits was two to eight times lower. The expression of IFN-α2 in leaves was dependent on the developmental stage of the leaf, with a decrease in IFN-α2 activity in fully developed leaves despite stable virus accumulation. This decrease can be attributed to a lower replication rate of the virus in mature tissues and a higher turnover of IFN-α2 compared to the stability of AGII virions. The highest level of IFN-α2 activity was much lower than expected based on the concentration of viral CP, likely due to instability or incorrect folding due to excess amino acid residues from cleavage sites flanking the insert [41].

In subsequent experiments, the *bar* gene encoding phosphinothricin acetyltransferase (PAT), whose expression in plants leads to the development of resistance to glufosinate ammonium-based herbicides, was inserted into the AGII vector. Infected plants acquired resistance approximately 7 dpi, with squash maintaining it for at least 26 days and cucumber up to 60 days. The AGII-Bar construct was ultimately tested on a sample of 450 plants representing various species of the *Cucurbitaceae* family, which were dusted with different doses of glufosinate ammonium 2 weeks after planting. Infection with the AGII-Bar construct protected the plants from the lethal effects of the herbicide even at the highest dose, leading to the complete removal of unwanted vegetation [42].

The AGII vector was eventually used for the expression of MAP30 and GAP31 proteins. These plant-derived proteins have antiviral, antitumor, and antimicrobial effects, making them potential candidates for therapeutic use. The prepared AGII-MAP30 and AGII-GAP31 constructs were infectious for all tested plant species, but symptoms (different from the parental AGII) were only exhibited in squash. Both proteins were successfully purified from infected squash leaves (designated as MAP30-SQ and GAP31-SQ) and subjected to characterization of their biological activity in vitro, showing identical effects to native MAP30 and GAP31. Purified MAP30-SQ and GAP31-SQ exhibited an inhibitory effect on HIV-1 infection by suppressing the expression of the viral core protein p24 and syncytium formation. An inhibitory effect on human herpesviruses 1 and 8 infections, as well as the growth of some pathogenic bacteria (*Staphylococcus aureus*, *Escherichia coli*), yeasts (*Candida albicans*), and microscopic fungi (*Aspergillus fumigatus*), was also demonstrated. Toxicity analysis of MAP30-SQ and GAP31-SQ showed no effect on the viability of human cells [43].

The backbone for the preparation of another expression vector based on ZYMV was the infectious clone p35SZYMV2-26, consisting of the genome of the Taiwanese isolate ZYMV TW-TN3 under the control of the CaMV 35S promoter. It was modified by introducing a cloning site between the *P1* and *HC-Pro* genes, as well as an additional cleavage site for NIa protease and a hexahistidine tag for C-terminal translational fusion with the target protein. The gene encoding the allergen group 5 of the house dust mite *Dermatophagoides pteronyssinus* (Der p 5), which is among the clinically significant allergens, was inserted into this modified ZYMV genome. Squash infected with the prepared construct exhibited systemic symptoms typical of the parental TW-TN3 virus, as well as a similar level of CP accumulation. The target protein Der p 5 was immunochemically detected in infected tissues in both free form and uncleaved fusion form with HC-Pro. The yield of Der p 5 was determined to be 1.5 μg per gram of leaf tissue. The construct was genetically stable over one year and 20 passages without the occurrence of deletion variants [44]. These results contradict previous observations in the case of inserting foreign sequences between the *P1* and *HC-Pro* genes, which were characterized by an increased frequency of recombination events and an impact on infection progression [13,26,38,41]. A concentrated lyophilized powder was prepared from the infected squash leaves and used to make an extract that was orally administered to mice in 10 daily doses, 7 days after prior intraperitoneal sensitization with the Der p 5 allergen. The protective effect was subsequently monitored after exposing the mice to Der p 5 allergen aerosol two and three weeks after sensitization. It was shown that in mice administered the extract, at least a 50% inhibition of Der-p-5-specific IgE antibody synthesis occurred compared to the control groups. Analysis of immune system cells present in the bronchoalveolar fluid indeed showed significantly lower numbers of eosinophils and neutrophils, indicating suppression of Der p 5 allergen-induced airway inflammation [44].

### 2.6. Turnip Mosaic Virus

Turnip mosaic virus (TuMV, *Potyvirus rapae*) is a significant pathogen of crops from the *Brassicaceae* family. As a backbone for constructing an expression vector based on TuMV, the infectious clone p35Tunos was used, which was modified by introducing cloning sites between the *P1* and *HC-Pro* genes or the *NIb* and *CP* genes. Reporter genes for GFP and GUS, including recognition sites for the respective viral proteases, were inserted into these regions. Infection of *Brassica rapa* var. perviridis plants with individual constructs led to the development of typical TuMV symptoms in both insertion sites. In the case of GFP, the symptoms were characterized by only a slightly delayed onset and a similar intensity compared to the wild-type virus. There were no differences in GFP accumulation depending on the insertion site, and both constructs were genetically stable for at least 30 days. In contrast, the GUS construct exhibited attenuated symptoms with a significantly delayed onset of the infection. The level of GUS expression was higher in the case of the NIb/CP insertion position, but spontaneous deletions in the insert region were observed in both constructs at 30 dpi. When cloning and simultaneously co-expressing both genes, the course of infection and genetic stability of the constructs were similar to those observed with GUS expression alone [45].

In another study, an infectious clone of TuMV (isolate YC5) was used to investigate the effectiveness and suitability of three novel insertion sites within the potyviral genome, positioned at the N-termini of the *P3*, *CI*, and *NIb* cistrons (i.e., between *HC-Pro*/*P3*, *6K1*/*CI*, and *NIa*/*NIb*, respectively). All three sites were proven to express both the GFP and Der p 5 encoding ORFs, but significant differences were observed in the genetic stability of the constructs and the course of infection. These differences were noted not only between individual insertion positions and inserted genes but also among different host plant species. Insertions at the *HC-Pro*/*P3* and *6K1*/*CI* sites were associated with delayed infection and milder symptoms in the tested host plants. In general, *Chenopodium quinoa* (local lesion host) and *N. benthamiana* (systemic host) provided the most consistent expression over multiple passages, remaining stable for up to 21 passages. On the other hand, *Brassica* hosts (systemic) exhibited varying stability, ranging from 13 (*B. campestris* var. *chinensis*) to 0 (*B. campestris* var. *pekinensis*) stable passages. The *6K1*/*CI* site had the lowest stability and expression efficiency among the three sites. The construct with the GFP insertion at this site frequently experienced deletions or reduced expression levels over multiple passages, particularly in systemic hosts. In the case of Der p 5 insertion, the construct failed to establish TuMV infection in all tested hosts; hence, the expression and stability of Der p 5 could not be assessed. The *HC-Pro*/*P3* insertion site demonstrated moderate stability and expression efficiency for both GFP and Der p 5 ORFs. While it was more stable than the *6K1*/*CI* site, it still showed a tendency to lose inserted genes over time, especially in systemic hosts. The *NIa*/*NIb* site demonstrated stable GFP expression but failed to produce detectable levels of Der p 5 in all *Brassica* hosts while showing a very weak detection signal in *N. benthamiana*. Overall, GFP was more efficiently expressed and stable across all conditions tested compared to Der p 5 [8].

To compare the effectiveness of these novel insertion sites with previously described insertion positions, constructs with GFP and Der p 5 ORFs inserted into the *P1*/*HC-Pro* and *NIb*/*CP* junctions were prepared in parallel. The *NIb*/*CP* site consistently provided the highest expression efficiency for both GFP and Der p 5 across all tested host plants. Interestingly, the *NIa*/*NIb* site exhibited higher GFP accumulation levels in several of the hosts compared to the *P1*/*HC-Pro* site. However, the latter demonstrated more consistent expression given the poor Der p 5 stability at the *NIa*/*NIb* site compared to the *P1*/*HC-Pro* junction [8].

### 2.7. Potato Virus A

Potato virus A (PVA, *Potyvirus atuberosi*) is a potyvirus that predominantly infects potatoes (*S. tuberosum*), but its experimental host range also includes other members of the *Solanaceae* family (including *Nicotiana* spp.). The PVA genome was first modified by inserting a foreign gene to study the role of the phosphorylation of PVA CP by CK2 kinase during viral infection. A full-length infectious cDNA clone of PVA was constructed, in which the reporter gene for GFP was inserted between the *NIb* and *CP* genes, flanked by cleavage sites for NIa-Pro. The prepared construct 35S-PVA-GFPNIb/CP was infectious and capable of both cell-to-cell and systemic spread, with successful expression of active GFP [46]. Subsequently, an expression vector PVA-(59) was derived from 35S-PVA-GFPNIb/CP by introducing unique restriction sites to simplify cloning. GFP and two human proteins—sorcin from the calcium-binding protein family and catechol-O-methyltransferase (S-COMT), which is involved in the metabolism of catechol estrogens and the degradation of the neurotransmitters dopamine and epinephrine—were inserted into PVA-(59). The prepared constructs were used to transfect *N. tabacum* and *N. benthamiana* plants, leading to systemic infection. Compared to the wild-type virus, the infected plants exhibited milder symptoms, with comparable or only slightly reduced accumulation of PVA CP. All constructs were genetically stable for at least 14–40 days post-infection. Plant infection led to successful expression of GFP as well as enzymatically active S-COMT. The estimated yield of S-COMT after affinity purification was approximately 0.9% of the total leaf protein (TLP) compared to an estimated PVA CP amount of approximately 2% TLP. The meta-methylation and para-methylation activity of plant-expressed S-COMT was 5% and 30% of the value compared to recombinant S-COMT expressed in *E. coli*, respectively, and the ratio of meta- to para-methylation of the substrate also differed. Differences in S-COMT enzymatic activity may be due to different physiological conditions between hosts for expression, such as pH, ion concentration, or the presence of plant compounds. In contrast to these observations, the expression of sorcin in plants at the protein level could not be detected despite ongoing infection, likely due to its instability in planta [47].

PVA was also used to characterize new potential insertion sites in the potyvirus genome for the expression of foreign genes. In previous experiments, a series of insertion mutants with random 15 bp insertions throughout the genome was prepared [48]. In selected viable mutants with insertions in the P1 (M14) and P3 (M45) cistrons, the entire or truncated gene encoding GFP (GFP/delGFP) was inserted, flanked by a recognition site for NIa-Pro at the 3′ end or both ends. While the M45 mutant lost infectivity, M14 chimeras with an insertion in the P1 cistron systemically infected *N. benthamiana* and *N. tabacum*. Symptoms of *N. benthamiana* infected with constructs for GFP expression were milder compared to the wild-type virus and without leaf malformation, but they exhibited a new symptom of vein clearing. Reduced pathogenicity correlated with decreased virus accumulation in systemically infected *N. benthamiana* leaves and *N. tabacum* protoplasts. Epifluorescence microscopy of infected plants and immunochemical analysis confirmed the expression of functional GFP, which, however, was not genetically stable. Deletion variants were detected in the primarily infected plants (18 dpi), and after the second mechanical passage, all tested samples showed the deletion genotype, with their progressive accumulation correlating with increasing infection symptom intensity, similarly to the wild-type virus [10]. These experiments were followed by attempts to simultaneously co-express three reporter genes from a single viral construct. Genes encoding GFP (G), luciferase (L), and GUS (U) were cloned into regions located between the P1/HC-Pro and the NIb/CP and at the 5′ end of the P1 cistron, respectively, to prepare a series of constructs with the insertion of one (pG00, p0L0, p00U), two (pGL0, pG0U, p0LU), or all three genes (pGLU). All constructs with the insertion of one or two genes were infectious, but with different symptom manifestations. While *N. benthamiana* infected with the p00U construct showed symptoms comparable to the empty vector and the wild-type virus, constructs p0L0, pG00, and p0LU caused only mild symptomatology. In the case of pGL0 and pG0U constructs, the absence of any infection symptoms was noted, and the plants were indistinguishable from the uninfected control group. RT-PCR analysis confirmed the intactness of the inserted segments in all tested plants for GFP and luciferase, but not for GUS, where most plants developed deletion genotypes or completely lost the insert. The pGLU construct systemically infected only two out of eight primarily infected plants, which showed no symptoms. After mechanical reinoculation of pGLU, infection developed in all plants. In this case, the inserted genes for GFP and luciferase remained intact during the observed period, while for GUS, partial or complete deletion of the insert occurred in two out of fifteen plants. Expression of target proteins in plants was recorded for all constructs, including pGLU, which increased the virus genome size by 39.2%. Infected plants were also subjected to electron microscopy analysis, which showed a direct correlation between the virus genome size and the resulting virion length [6].

### 2.8. Soybean Mosaic Virus

Soybean mosaic virus (SMV, *Potyvirus glycitessellati*) is a globally widespread pathogen of soybean, but its experimental host range also includes members of the genera *Pisum*, *Phaseolus*, and *Nicotiana* [49]. The expression vector was based on its cDNA clone SMV-G7H, which was modified by introducing cloning sites and an additional recognition site for NIa protease between the *P1* and *HC-Pro* genes. The reporter gene for GFP was initially cloned into the resulting vector pSMV-MCS. In infected soybean plants, typical mosaic symptoms of infection comparable to the parental virus developed, and the insertion of the foreign sequence also did not affect virus accumulation. The emission of green fluorescence in the upper leaves of infected plants indicated systemic accumulation of GFP. The construct was also genetically stable over three monitored mechanical passages of the virus. Subsequently, genes encoding viral suppressors of gene silencing, CMV 2b and TBSV p19, were cloned into pSMV-MCS, whose expression should lead to more severe SMV infection symptoms. Indeed, infected plants developed intense infection symptoms, including stunting and leaf deformation, with virus accumulation comparable to the wild-type SMV. Finally, pSMV-MCS was studied for its use as a vector for virus-induced gene silencing (VIGS) by inserting partial sequences of the phytoene desaturase (PDS) gene in both direct and reverse orientations [50]. PDS is an enzyme responsible for carotenoid production, and its reduced expression leads to loss of leaf pigmentation [51,52]. However, infected plants did not show signs of silencing the expression of PDS despite the stable insertion of the sequences. This result was not unexpected given the presence of the potyviral HC-Pro protein, which is a strong suppressor of gene silencing [50].

In subsequent experiments, pSMV-MCS was modified to enable simultaneous co-expression of two genes by inserting a second insertion cassette between the *NIb* and *CP* genes, consisting of a cloning site and an additional recognition site for NIa-Pro. To prevent virus transmission between plants, a substitution mutation of threonine to alanine in the PTK motif of HC-Pro, essential for its aphid transmission, was also performed. The resulting vector pSMV-Dual was infectious, and biological experiments demonstrated that unlike the parental pSMV-G7H and pSMV-MCS, it is not aphid-transmissible. Subsequently, genes for GFP and cyan fluorescent protein (CFP) were inserted into pSMV-Dual. To specifically distinguish the individual proteins based on different subcellular localization, CFP was expressed in fusion with a nuclear localization signal derived from the SV40 T antigen. The prepared construct systemically infected soybean plants, and confocal microscopy demonstrated GFP accumulation in the cytoplasm, nucleoplasm, and plasma membrane of infected cells, while the fluorescent signal corresponding to CFP was observed exclusively in the nucleus. The insertion of both genes was stable over three monitored serial mechanical passages of the virus [53].

Based on these findings, the pSMV-Dual vector was subsequently applied to visualize protein–protein interactions in soybeans using bimolecular fluorescence complementation (BiFC) [53].The principle of BiFC involves the co-expression of two studied proteins fused with non-functional N- or C-terminal halves of a fluorescent protein. In the case of mutual interaction of these proteins, the functional form of the fluorescent protein is restored, resulting in a fluorescent signal [54,55]. The gene encoding the B2 protein of Flock House Virus (FHV), known to interact with itself, was inserted into pSMV-Dual in fusion with fragments of yellow fluorescent protein (YFP). A strong fluorescent signal was observed in infected plants, indicating specific mutual interaction between copies of the B2 protein [53].

Finally, the pSMV-Dual vector was applied to identify interacting protein partners through co-immunoprecipitation [53]. This method is based on the expression of the studied protein in fusion with a short epitope tag (e.g., FLAG). The resulting protein complexes are then precipitated using an antibody specific to the used tag and analyzed through mass spectrometry [56]. The potyviral protein HC-Pro plays a key role at many levels of infection and is likely to interact with several viral and cellular proteins [57]. Its oligomerization and interaction with potyviral CP have been demonstrated [58,59]. To confirm known interactions and identify new protein partners, the gene for HC-Pro in N-terminal fusion with a FLAG tag was inserted into pSMV-Dual. Analysis of immunoprecipitates from infected plants confirmed the formation of dimeric and tetrameric forms of HC-Pro and its interaction with SMV CP. Interaction of HC-Pro with two cellular proteins—glyceraldehyde-3-phosphate dehydrogenase and subunit IV of the cytochrome b6/f complex—was also identified. These experiments highlight the robust applicability of potyviral vectors not only in biotechnological applications but also in the study of protein–protein interactions in planta [53].

### 2.9. Tobacco Vein Banding Mosaic Virus

Tobacco vein banding mosaic virus (TVBMV, *Potyvirus nicotianavenaobscurum*) is a virus that primarily infects plants belonging to the *Solanaceae* family, including several species of the genus *Nicotiana*. For the purposes of heterologous expression, a full-length cDNA clone of its isolate HN39 was used. This clone was constructed by introducing the 35S promoter of CaMV and three introns into the region of the P3 and CI cistrons to ensure the stability of the viral genome in the plasmid. The gene encoding GFP was inserted between the *NIb* and *CP* genes in the resulting clone. The prepared construct infected *N. benthamiana*, which exhibited symptoms similar to the parental virus. Strong fluorescence corresponding to a high level of GFP accumulation was observed in systemically infected leaves at 14 dpi [60].

### 2.10. Sugarcane Mosaic Virus

Sugarcane mosaic virus (SCMV, *Potyvirus sacchari*) is an important pathogen of sugarcane, maize, and other grass species, causing a disease known as sugarcane mosaic. To achieve the expression of foreign proteins in maize, an infectious cDNA clone of SCMV, derived from the MDMV-B isolate, was constructed. The SCMV genome was placed under the control of the CaMV 35S promoter and the nopaline synthase terminator. The resulting clone was then modified by introducing two different cloning sites, as well as an additional recognition site for NIa protease between the *P1* and *HC-Pro* genes. Transmissibility of the virus by aphids was prevented by mutagenesis of the DAG motif in the capsid protein, replacing alanine with threonine. Based on the cloning site, resulting vectors were designated pSCMV-CS1 and pSCMV-CS2, respectively. To evaluate its potential for protein expression in maize, the GFP-encoding sequence was cloned into the pSCMV vectors. Two weeks post-inoculation, typical mosaic symptoms were observed on the leaves of infected plants. Using fluorescent microscopy, green fluorescence was detected in symptomatic leaves, which was prominent in lighter green to yellow areas of the leaf. GFP expression was consistent throughout the entire length of the leaf. The intact GFP insert was detected in most samples even after two months of cultivation, although some minor deletions were found in several upper leaf samples. To further evaluate the ability of SCMV to express functional proteins of varying sizes, the genes *uidA* and *bar* encoding GUS and PAT were inserted into the pSCMV vectors. All constructs were infectious, and the expression of functional proteins was confirmed. GUS activity was detected throughout the leaves, with no background activity in control plants infected with empty vector. In the case of PAT-expressing plants, resistance to the herbicide Finale^®^ was observed, and all infected plants survived the treatment. These results demonstrated that the modified SCMV vector can successfully express different biologically active proteins of foreign origin. Moreover, infection with the pSCMV vector was successfully established in the B73 dent corn inbred line, demonstrating that SCMV expression vectors can be effectively used across a wide range of maize genetic backgrounds, making them a versatile tool for maize research [61].

### 2.11. Zinnia Mild Mottle Virus

Zinnia mild mottle virus (ZiMMV) is a recently discovered potyvirus that was found in common zinnia plants (*Zinnia elegans*) displaying mild leaf mottling and flower curling symptoms. A full-length infectious cDNA clone pCB-ZiMMV was used for the expression of several foreign proteins in common zinnia and other *Asteraceae* plants: a fragment of enhanced GFP (eGFP), GUS, FLAG-tagged Cas9 protein, ZeMYB9 transcription factor, and gibberellin-20-oxidase. Heterologous sequences were inserted between *NIb* and *CP* cistrons and flanked by NIa-Pro cleavage sites. The eGFP gene fragment was successfully inserted and expressed in common zinnia plants, developing typical symptoms of systemic ZiMMV infection approximately 10 dpi. All infected plants exhibited prominent green fluorescence in both systemic leaves and flowers. The eGFP-encoding sequence remained detectable even after three mechanical passages, confirming the genetic stability of the construct. Similarly, the vector enabled stable systemic expression of GUS in both stem and root tissues. In contrast, the insertion of the 4266 nt long gene encoding Cas9 protein prevented the systemic spread of ZiMMV infection, and the Cas9 expression was restricted to inoculated leaves. Based on these results, authors attempted to use this expression vector as a tool for functional studies in common zinnia by inserting the gene encoding ZeMYB9 transcription factor [62]. Overexpression of ZeMYB9 induces a dark red color in flowers and leaves due to the elevated levels of antocyanins [63]. Plants transfected with this construct indeed exhibited dark red speckles and 10-fold higher antocyanin accumulation compared to mock and wild-type ZiMMV-infected plants, demonstrating the usefulness of this system [62]. Moreover, the pCB-ZiMMV vector was used for the expression of the gene *AtGA5* encoding gibberellin-20-oxidase as the overexpression of this gene has been shown to promote cell elongation and morphogenesis [64,65]. Transfected plants indeed showed a significant increase in height (29%) compared to control plants. The effectiveness of the pCB-ZiMMV vector was further tested by infecting other species within the *Asteraceae* family with the construct for eGFP expression (Table 1). Among eight tested species, five developed systemic ZiMMV infection and exhibited green fluorescence in the upper leaves. In the case of *Helianthus annuus*, the infection and eGFP accumulation remained localized in the infiltrated leaves. The remaining three species did not develop infection at all [62].

## 3. Potyviruses as Carriers of Foreign Antigens

Like many other groups of viruses, potyviruses have been used to prepare chimeric virus particles presenting foreign peptides on their surfaces (Table 2). The first vector constructed for the fusion of foreign peptides with potyvirus CP was pGPPV-NATMluI, derived from the infectious clone pGPPV [25], which was modified by deleting 45 nt in the region encoding the N-terminus of CP, described in naturally aphid-nontransmissible (NAT) PPV mutants. A unique MluI restriction site was introduced into the deletion site for cloning purposes. A sequence encoding a 15-amino-acid-long epitope from the VP2 protein of canine parvovirus (CPV) was inserted into pGPPV-NATMluI either as a single copy or in a double tandem repeat. Both constructs systemically infected *N. clevelandii* with unchanged symptomatology, and the accumulation and stability of the chimeric virions of both types were comparable to wild-type PPV particles. Immunochemical analysis showed a strong specific reaction with a monoclonal antibody against the corresponding CPV epitope. The immunogenicity of purified chimeras was confirmed in both mouse and rabbit models, including virus-neutralizing activity of sera after immunization [66]. The lower tolerance of pGPPV-NATMluI to insertions of other peptides led to efforts to identify new potential insertion sites within the N-terminal domain of CP. Using PEPSCAN analysis of PPV CP, fragments reacting with sera from immunized animals were identified, and it was shown that the immunogenicity of foreign fused peptides in vivo was closely related to the inherent immunogenicity of the given PPV CP region, determined by its degree of surface accessibility in the native structure of the virion [11].

The AGII vector based on ZYMV was also used for epitope presentation. Initially, sequences encoding a hexahistidine tag (AGII-6xhis) and a 16 amino acid peptide from the human c-Myc protein (Myc) were inserted into AGII in an N-terminal translational fusion with CP. Both chimeric viruses were systemically infectious and genetically stable, and their accumulation in cucurbits was comparable to the parental AGII. Immunochemical analysis showed specific reactivity of both chimeras with antibodies against the fused peptides, but the weakened detection signal with the ZYMV CP antibody was observed, likely due to the masking of its antigenic determinants. Successful purification of AGII-6xhis through affinity chromatography under native conditions confirmed the exposure of the fused peptide on the virion surface. CP fusion with Myc was also used to label AGII deletion mutants prepared to identify non-essential parts of the N-terminal domain of ZYMV CP, which are dispensable during systemic infection. It was found that even the removal of 33 amino acids did not lead to a reduction in infectivity or symptoms compared to AGII, and approximately one-third of the infectivity was retained even after the deletion of the entire N-terminal domain of CP (43 amino acids). Unlike Myc, inserting an equally long foot-and-mouth disease virus (FMDV) CP-derived peptide into one of the AGII deletion mutants resulted in the loss of virus infectivity, which was surprisingly restored by N-terminal fusion of this peptide with Myc [67]. These observations were later clarified by experiments demonstrating a direct dependence of ZYMV infectivity on maintaining the naturally neutral charge of the N-terminal domain of CP. Insertion of a highly basic FMDV peptide increased its charge, which was subsequently neutralized by inserting the negatively charged Myc [68].

Among the more recent examples of using potyviruses as carriers of foreign antigens is the p35Tunos vector based on TuMV. In this vector, a segment encoding a 20 amino acid peptide derived from the human vascular endothelial growth factor receptor 3 (VEGFR3) was inserted into an N-terminal translational fusion with CP. The resulting construct pVER3 systemically infected *A. thaliana* and *Brassica juncea*, in which it was genetically stable during two monitored mechanical passages. Purified pVER3 particles specifically reacted with an antibody against the peptide, and immunoelectron microscopy confirmed its presence on the virion surface. Chimeric pVER3 virions were then subjected to in vivo immunogenicity tests. Sera from mice immunized with pVER3 showed significantly higher titers of specific antibodies compared to the group immunized with an equivalent amount of the unfused peptide. When pVER3 particles were used as immobilized antigen in ELISA tests, up to 30-fold higher detection sensitivity was observed based on reactivity with reference serum [69]. In a follow-up study, a segment encoding a 14 amino acid peptide from the thrombin receptor was inserted into p35Tunos in fusion with TuMV CP. However, unlike pVER3, there was a significant reduction in virus infectivity, and attempts to restore it by modifying the amino acid context of the inserted peptide were unsuccessful [70].

The latest example is the production of plant-derived nanoparticles decorated with nanobodies by using ZYMV and TEV-based vectors [71]. Nanobodies are an interesting alternative to monoclonal antibodies consisting of the variable domain of heavy chain (VHH) antibodies from animals belonging to the family *Camelidae*. With molecular masses of 12–15 kDa, nanobodies are the smallest currently known antigen-binding proteins, making them attractive therapeutic molecules [72]. To explore the potential of ZYMV as a platform for displaying larger moieties, such as nanobodies, the vector AGII-Δ33 was used. As demonstrated previously by [67], this construct tolerated the replacement of 33 amino-terminal moieties of the CP with heterologous peptides without a significant impact on virus infectivity. The sequence encoding anti-αGFP nanobody was cloned into the AGII-Δ33 to produce fully decorated nanoparticles. To prepare partially decorated nanoparticles, a derivative clone containing a picornavirus splicing domain F2A between the nanobody and the CP was also constructed. Zucchini plants agroinoculated with these recombinant viruses displayed varying symptoms; while the F2A containing construct caused mild symptoms, the clone without an F2A domain produced no visible symptoms. RT-PCR analysis indeed confirmed the presence of the nanobody sequence only in plants infected with F2A-containing virus. These results suggest that the F2A cleavage system allowed for the production of partially decorated viral particles and the stable expression of the nanobody, making ZYMV a promising platform for displaying functional proteins on viral particles. Chimeric virions were purified from zucchini plants, and their structural integrity was verified through transmission electron microscopy (TEM). Further analyses confirmed successful GFP binding activity [71].

Similarly, the anti-αGFP-encoding sequence was cloned into the TEV-based vector either with or without a self-cleaving F2A domain. Unlike ZYMV, the foreign sequence was inserted into the TEV genome without deleting any region of the CP coding sequence. Surprisingly, unlike the results with ZYMV, the clone without F2A caused symptoms of systemic infection following agroinoculation of *N. benthamiana* plants. RT-PCR analyses confirmed the anti-αGFP nanobody sequence in viral progenies, and Western blot showed the expression of the fused CP–nanobody chimeras. Purification of the chimeric particles showed a decreased yield for recombinant TEV compared to the wild-type virus, but TEM analysis confirmed the presence of typical TEV particles. Further analyses proved the ability of purified particles to bind GFP, confirming that the nanobodies remained functional after virion assembly. These results demonstrated the successful production of functional nanobody-decorated viral nanoparticles using TEV-based recombinant clones [71].

The TEV-based vector was further applied for the production of plant-derived nanoparticles decorated with nanobodies recognizing two different regions of the receptor-binding domain (RBD) of the SARS-CoV-2 Spike protein (designated as VHH1 and VHH2, respectively). Initially, the VHH1-encoding cDNA was cloned into the TEV infectious clone to achieve N-terminal fusion with TEV CP. Three additional derivatives of this construct were also created, incorporating different 2A self-cleaving peptides (P2A, E2A, and F2A) exhibiting various cleavage efficiencies to modulate the ratio of decorated (nanobody-fused) versus free CP. The construct without a 2A peptide was fully decorated with nanobodies and maintained infectivity, while the addition of 2A peptides enabled mixed populations of decorated and free CP within the virions. Interestingly, the TEV-VHH2 construct was non-infectious, indicating that the viability of fully decorated VNPs depends on the nanobody itself. However, adding an F2A peptide restored infectivity, resulting in partially decorated particles. The research proceeded to purify VNPs from *N. benthamiana* plants infected with TEV-VHH1 or TEV-VHH2-F2A, verifying the presence of both decorated and free CP. Quantification showed that VHH1-CP and VHH2-F2A-CP accumulated to approximately 25 and 50 μg per gram of fresh tissue, respectively. Immunoelectron microscopy confirmed the successful decoration of the VNPs, as seen through gold-labeled staining around the VHH-decorated particles, which was absent in the wild-type control. ELISA tests demonstrated that TEV-derived VNPs, decorated with either VHH1 or VHH2, effectively recognized the RBD, confirming the antigen-binding activity of the VNPs. In a pseudovirus neutralization assay, VHH-decorated VNPs displayed dose-dependent neutralization activity against a GFP-expressing pseudotyped virus, with nearly complete inhibition at higher concentrations. TEV-derived VNPs carrying VHH1 showed an IC50 of 0.4 μg/mL. This performance was notably better than a dimeric VHH1 control, suggesting that multivalency on the VNPs enhances neutralizing efficiency [73].

In our laboratory, the pAD expression vector was modified for the fusion of foreign peptides with PPV CP by removing the viral protease recognition site. Although the resulting vector pADep (referred to as pBOR-VTep in the older literature) was infectious, inserting most tested foreign sequences led to the loss of virus viability. A successful example of pADep utilization is the preparation of chimeric virions presenting an 18 amino acid peptide derived from IAV HA. However, inserting another HA IAV fragment of the same length resulted in the loss of construct infectivity (Achs and Šubr, unpublished results). Previous experiments on ZYMV suggested the role of the overall net charge of the N-terminal domain of the capsid protein in systemic virus infectivity [68]. Surprisingly, maintaining the naturally negative net charge of the N-terminal domain of PPV CP did not restore the viability of these chimeras, suggesting the role of different factors that determine virus infectivity. Our results show that the very end of CP PPV is not suitable for the insertion of foreign peptides (Achs and Šubr, unpublished results). However, internal positions located within the N-terminal domain might be eligible for this purpose, as suggested by previously published results [11]. Moreover, using self-cleaving protein domains, such as F2A, has proven a useful strategy to produce viable chimeras [72].

**Table 2:** A summary of potyviruses that have been applied for displaying foreign peptides and epitopes on the surfaces of potyviral particles through their fusion with the capsid protein. For each potyvirus, the table summarizes the fusion position of a foreign peptide, the target peptide, and the host plants used for the experiments.

Virus Species	Fusion Position	Target Peptide	Host Plants	Reference
Plum pox virus	several internal fusion positions within the N-terminal domain of the CP	epitope from VP2 CPV	*Nicotiana clevelandii*	[11,66]
	extreme N-terminus of the CP	peptide from HA IAV	*Nicotiana benthamiana*	Achs and Šubr, unpublished results
Zucchini mosaic virus		hexahistidine tag epitope from human c-Myc epitope from CP FMDV	*Cucurbita pepo* *Cucumis sativus* *Cucumis melo*	[67]
		anti-αGFP nanobody	*Cucurbita pepo*	[72]
Turnip mosaic virus		epitope from human VEGFR3	*Arabidopsis thaliana* *Brassica juncea*	[70]
Tobacco etch virus		anti-αGFP nanobody	*Nicotiana tabacum*	[72]
		anti-SARS-CoV2 Spike protein RBD domain nanobodies	*Nicotiana benthamiana*	[73]

## 4. Conclusions

**Figure 2:**
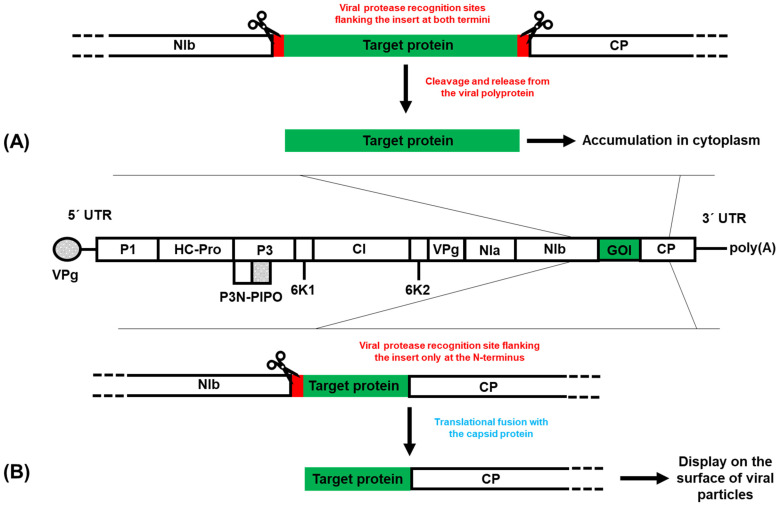
Graphical summary of two main strategies for using potyvirus-based expression vectors. In the first strategy (**A**), the target protein is flanked by recognition sites for a viral protease (red boxes) at both termini, allowing for its cleavage and release from the viral polyprotein during infection. In the second strategy (**B**), the target protein is translationally fused to the viral capsid protein with a single protease recognition site at the N-terminus, resulting in chimeric viral particles that display the protein on their surface. GOI—gene of interest.

Potyvirus-based expression vectors offer distinct advantages for heterologous protein expression in plants, such as efficient polyprotein expression, flexibility in insertion sites, and a broad host range. These vectors allow for high-yield protein production, with options for either free or fused expression (Figure 2), which can be tailored to specific applications. Potyviruses are especially promising for expression in a wide range of plants, including edible crops, providing potential for accessible vaccine delivery or therapeutic protein production. Several studies have demonstrated the versatility of potyvirus-based vectors for the expression of foreign recombinant proteins or the production of chimeric virus particles that present foreign peptides on their surfaces. Such constructs have shown promising results in eliciting immune responses in animal models, highlighting their potential for vaccine development [44,66]. The possibility of using multiple insertion sites enables the simultaneous expression of multiple proteins from a single construct, supporting complex metabolic engineering and the coordinated production of multi-subunit proteins [6,19,21].

However, several limitations accompany these benefits. The stability of the inserted genes can be inconsistent, with larger genes or certain insertion sites prone to deletions over time, particularly after multiple passages [6,9,13,14,26,28,38,41,45]. Therefore, although potyviruses support high protein yield, their capacity is generally restricted to smaller transgenes. Expression efficiency and stability may also vary among hosts, with unwanted symptoms or limited systemic spread in some plants impacting their overall utility.

Finally, the potyviral capsid protein can only tolerate small antigen fusions, as larger or structurally complex epitopes often disrupt particle assembly or impair the virus’s ability to spread systemically within the plant host [67,72]. To maintain infectivity of these chimeras, several strategies have been developed, including the use of self-cleaving peptides to produce both fused and native CP copies, as well as adjusting the net charge of the CP by adding specific residues [68,72,73].

Despite these challenges, with careful optimization of the gene size, the insertion site, and the host, the potential applications of potyviruses in biotechnology are vast, as shown by the latest studies [62,72,73]. They offer a robust platform for the expression of foreign proteins, the study of protein interactions, and the development of novel vaccines. Ongoing research and modifications to these vectors continue to enhance their efficiency and broaden their applicability, paving the way for innovative solutions in plant biotechnology and beyond.

## Figures and Tables

**Table 1:** A comprehensive summary of various potyviruses that have been employed as vectors for heterologous gene expression in plants. For each potyvirus, the table summarizes the insertion position of a foreign gene, target foreign proteins, and the host plants used for the experiments.

Virus Species	Insertion Position	Product of Expression	Host Plants	Reference
Tobacco etch virus	*P1*/*HC-Pro*	GUS	*Nicotiana tabacum*	[13,15]
		CP BYV HSP70h BYV P20 BYV L-Pro BYV	*Nicotiana tabacum* *Nicotiana benthamiana*	[18]
	*NIa-Pro*/*CP*	mCherry Venus BFP	*Nicotiana tabacum* *Nicotiana benthamiana*	[12]
		Metabolic enzymes from *Pantoea ananatis*	*Nicotiana tabacum*	[21]
	*NIa-Pro*/*CP* 5′ UTR/*P1*	GFP mCherry	*Nicotiana tabacum*	[9]
Plum pox virus	*P1*/*HC-Pro*	GUS	*Nicotiana clevelandii*	[26]
	*NIb*/*CP*	GFP VP60 RDHV	*Nicotiana clevelandii*	[28]
		GFP DsRed	*Nicotiana benthamiana*	[30]
		PB1-F2 IAV	*Nicotiana benthamiana*	[34]
		sHSP from *Cronobacter sakazakii* SARS-CoV-2 nucleocapsid protein and its fragments	*Nicotiana benthamiana* *Papaver somniferum*	[35]
Clover yellow vein virus	*P1*/*HC-Pro*	GFP Soybean glutamine synthetase	*Vicia faba* *Phaseolus vulgaris* *Glycine max* *Pisum sativum* *Nicotiana benthamiana*	[36]
Lettuce mosaic virus	*P1*/*HC-Pro*	GFP GUS	*Lactuca sativa*	[38]
Zucchini yellow mosaic virus	*NIb*/*CP*	CP CMV GFP GUS IFNα-2	*Cucurbita pepo* *Cucumis sativus*	[41]
		PAT	*Cucurbita pepo* *Cucumis melo* *Cucumis sativus*	[42]
		MAP30 GAP31		[43]
	*P1*/*HC-Pro*	GFP Der p 5	*Cucurbita pepo*	[44]
Turnip mosaic virus	*P1*/*HC-Pro* *NIb*/*CP*	GFP GUS	*Brassica rapa* var. perviridis	[45]
	*HC-Pro*/*P3* *6K1*/*CI* *NIa*/*NIb* *P1*/*HC-Pro* *NIb*/*CP*	GFP Der p 5	*Chenopodium quinoa* *Nicotiana benthamiana* *Brassica juncea* *Brassica campestris* (var. *chinensis*, *ching-geeng*, *pekinensis*)	[8]
Potato virus A	5′ terminus of *P1* cistron	GFP	*Nicotiana tabacum* *Nicotiana benthamiana*	[10]
	*NIb*/*CP*	GFP		[46]
		GFP S-COMT		[47]
	*NIb*/*CP* *P1*/*HC-Pro* 5′ terminus of *P1* cistron	GFP GUS Luciferase		[6]
Soybean mosaic virus	*P1*/*HC-Pro*	GFP P19 TBSV 2b CMV	*Glycine max*	[50]
	*P1*/*HC-Pro* *NIb*/*CP*	GFP CFP YFP B2 FHV HC-Pro		[53]
Tobacco vein banding mosaic virus	*NIb*/*CP*	GFP	*Nicotiana benthamiana*	[60]
Sugarcane mosaic virus	*P1*/*HC-Pro*	GFP GUS PAT	*Zea mays*	[61]
Zinnia mild mottle virus	*NIb*/*CP*	GFP GUS ZeMYB9 Gibberellin-20-oxidase	*Zinnia elegans* *Zinnia baageana* *Helianthus annuus* *Callistephus chinensis* *Taraxacum mongolicum* *Matricaria chamomilla*	[62]
